# 3D Printed, PVA–PAA Hydrogel Loaded-Polycaprolactone Scaffold for the Delivery of Hydrophilic In-Situ Formed Sodium Indomethacin

**DOI:** 10.3390/ma11061006

**Published:** 2018-06-13

**Authors:** Mershen Govender, Sunaina Indermun, Pradeep Kumar, Yahya E. Choonara, Viness Pillay

**Affiliations:** Wits Advanced Drug Delivery Platform Research Unit, Department of Pharmacy and Pharmacology, School of Therapeutic Sciences, Faculty of Health Sciences, University of the Witwatersrand, 7 York Road, Parktown, Johannesburg 2193, South Africa; mershen.govender@wits.ac.za (M.G.); sunaina.indermun@wits.ac.za (S.I.); pradeep.kumar@wits.ac.za (P.K.); yahya.choonara@wits.ac.za (Y.E.C.)

**Keywords:** 3D printing, polycaprolactone scaffold, PVA–PAA hydrogel, sodium indomethacin delivery, stress-strain testing, viscoelastic analysis

## Abstract

3D printed polycaprolactone (PCL)-blended scaffolds have been designed, prepared, and evaluated in vitro in this study prior to the incorporation of a polyvinyl alcohol–polyacrylic acid (PVA–PAA) hydrogel for the delivery of in situ-formed sodium indomethacin. The prepared PCL–PVA–PAA scaffold is proposed as a potential structural support system for load-bearing tissue damage where inflammation is prevalent. Uniaxial strain testing of the PCL-blended scaffolds were undertaken to determine the scaffold’s resistance to strain in addition to its thermal, structural, and porosimetric properties. The viscoelastic properties of the incorporated PVA–PAA hydrogel has also been determined, as well as the drug release profile of the PCL–PVA–PAA scaffold. Results of these analyses noted the structural strength, thermal stability, and porosimetric properties of the scaffold, as well as the ability of the PCL–PVA–PAA scaffold to deliver sodium indomethacin in simulated physiological conditions of pH and temperature. The results of this study therefore highlight the successful design, fabrication, and in vitro evaluation of a 3D printed polymeric strain-resistant supportive platform for the delivery of sodium indomethacin.

## 1. Introduction

Tissue engineering applies the principles of both engineering and sciences to produce bio-mimetic three-dimensional (3D) scaffolds with therapeutic applications [1]. The properties of scaffolds are unique in which they allow for the ability to assist in cellular growth and regeneration whilst simultaneously providing mechanical and structural support [2]. In order to produce a successful 3D scaffold, it has been noted a well-defined interconnected pore network for cell growth and flow transport of nutrients and metabolic waste is essential to achieving successful application as well as the possession of mechanical properties mimicking those of the tissues at the implantation site [3,4,5].

Polycaprolactone (PCL) is an extensively used synthetic, biocompatible polymer in the biomedical field. Displaying superior rheological and viscoelastic properties, this hydrophobic polymer has been utilized in many devices and implants [4,5]. This slow-degrading polymer renders is suitable for long-term delivery and structural support as the biodegradation of PCL is slow in comparison to other polymers. Desired release profiles and degradation profiles are also made possible with the use of PCL, as it has the ability to form compatible blends with other polymers [6]. The versatility of PCL has stimulated extensive research into its potential application in 3D tissue engineering [7]. Recent studies have determined the potential of PCL (Mw 48,000–90,000) as a suitable material for cartilage tissue replacement [8,9,10]. These studies have also noted the formation of robust matrices with increased resistance to strain. The positive results achieved therefore allow for PCL to be potentially utilized as a reinforcement scaffold for load-bearing biological tissues such as ligaments and tenons. The primary concern however with the use of PCL (Mw 48,000–90,000) in 3D printing is the viscous, robust nature of the melted polymer, requiring slow printing under high pressure. This study seeks to improve on this 3D printing methodology by developing a PCL blend of varying molecular weights to allow for a more effective 3D printing process.

Additionally, recent studies have also been conducted in addressing an optimal scaffold architecture with the aim of achieving efficient biomechanical performance and tissue formation in vivo [11,12]. This would allow for the development of an effective support system that would promote healing of the damage biological tissue. Pore size, as well as pore interconnection, are also proposed as a few key parameters seen as pertinent to model the ideal scaffold architecture, although no parameters have been clearly defined [13,14,15]. In addition to the mechanical and structural considerations, the application of drug delivery systems in these scaffolds requires the use of various polymers that may be utilized to achieve the desired physicochemical properties, characteristics, and drug release profile. This property in addition to structural support would ensure a pharmacological component to the scaffold system to allow for greater healing of the damaged tissue. This ultimately will allow for the development of a functional, effective, reproducible 3D printed system for enhanced tissue healing through drug delivery application. 

Such a scaffold system could be highly beneficial in conditions such as ligament and tendon damage, which often results in residual inflammation, with the administration of adjunctive anti-inflammatory agents often being a requirement. The addition of anti-inflammatory agents to the 3D printed structural support scaffolds, which can be sutured onto the surgically repaired load-bearing tissue, can therefore allow for a more holistic treatment system that will allow for acute treatment of site-specific inflammation, whilst allowing for long-term structural support to allow for adequate tissue repair and rehabilitation. In this study, the sodium salt of indomethacin, a non-steroidal anti-inflammatory drug (NSAID) indicated for the treatment of rheumatoid arthritis, severe pain, and acute post-operative pain treatment serves as the candidate drug. The sodium salt of indomethacin was utilized due to the poor hydrophilicity of the parent indomethacin molecule. The drug has been incorporated into the architecture of the PCL scaffold by means of a semi-interpenetrating network (IPN) composed of polyvinyl alcohol (PVA) and polyacrylic acid (PAA). The semi-IPNs will allow for the production of a relatively dense hydrogel matrix whilst strengthening mechanical properties and providing more efficient drug loading in comparison to conventional hydrogels [16]. A comprehensive in vitro characterization of the 3D printed PCL scaffold and PVA–PAA hydrogel has been undertaken in addition to sodium indomethacin release analysis under simulated physiological conditions of pH and temperature. Characterization techniques that have been utilized include calorimetrical and porosimetric measurements of the PCL blend, viscoelastic analysis of the PVA–PAA hydrogel, and strain analysis and magnetic resonance imaging of the PCL–PVA–PAA scaffold. 

## 2. Materials and Methods

### 2.1. Materials

Polycaprolactone (Mn 10,000), n-Polycaprolactone (Mw 70,000–90,000, typical Mn = 80,000), Indomethacin (≥99%), Poly(vinyl alcohol) (Mw 89,000–98,000, 99+% hydrolyzed), Acrylic acid (anhydrous, 99%) and *N*,*N*-Methylenebisacrylamide (≥99.5%) were purchased from Sigma Aldrich (St Louis, MI, USA). All other chemicals were of analytical grade and were used as received.

### 2.2. Preparation of the Polycaprolactone Blend

The Polycaprolactone blend was prepared by incorporation of PCL (Mn 10,000; PCL_10_) and *N*-Polycaprolactone (Typical Mn 80,000; PCL_80_) at a ratio of 40:60 (PCL_10_:PCL_80_). This ratio was chosen to be printed due to its decreased viscosity, allowing for a more efficient printing process in addition to a robust structure after cooling to room temperature. For the preparation of the PCL blend, 50 g of the chosen combination was added to a glass beaker and heated on a magnetic stirrer at 100 °C until homogenous. The molten PCL-blend was thereafter placed onto laboratory film and allowed to cool to room temperature before 3D printing and evaluation.

### 2.3. Design and Printing of the PCL-Blended Scaffold

**Figure 1:**
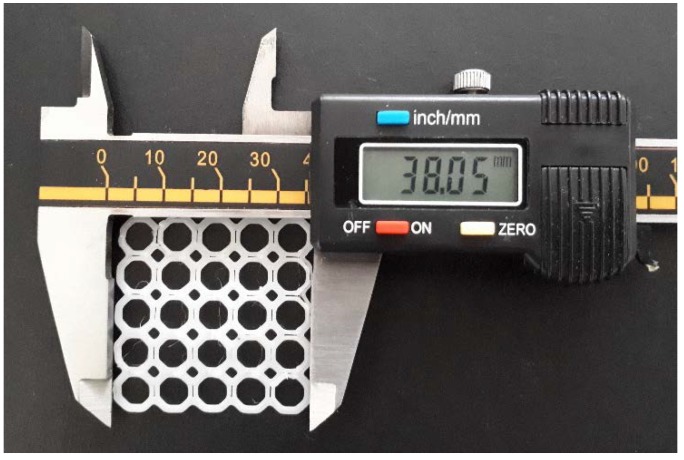
Digital image of the 3D printed PCL scaffold measured with a digital caliper.

The PCL-blended scaffold was designed using Magics^®^ V18 design software equipped with Envisiontec^®^ printing protocols and 3D printed using an Envisontec^®^ 3D Bioplotter (Envisontec^®^ GmbH, Gladbeck, Germany) equipped with high and low temperature printing heads. After insertion of the prepared PCL blend into the high temperature printing head, the printing head was heated to 130 °C and allowed to equilibrate for 15 min. The PCL scaffold was thereafter printed as per the design protocol at a pressure of 6.0 bar, a printing speed of 2.5 mm/s and a pre-flow and post-flow timing of 0 s (Figure 1). The octagonal orientation was chosen from additional geometrical orientations, due to its robustness and resistance to strain (Supplementary Materials).

### 2.4. Characterization of the PCL Blend

#### 2.4.1. Calorimetrical Measurements of the PCL Blend

Thermal analysis of the PCL blend and its polymeric constituents was undertaken employing a differential scanning calorimeter (DSC; Mettler Toledo DSC-1 STARe System, Schwerzenback, ZH, Switzerland). Samples (8 mg) were placed within sealed aluminum pans and subjected to ramped heating at 10 °C/min at a temperature range of 25–200 °C under a constant purge of an inert N_2_ atmosphere (100 mL/min). Thermal transitions were assessed and identified from the resultant DSC thermal profiles, corresponding to the total heat flow (ΔH) achieved. Temperature modulated differential scanning calorimetry (TMDSC) was further carried out on the PCL blend to identify its thermal characteristics at a temperature range of 25–65 °C, the temperature range where all thermal events were displayed in the DSC analysis. The sinusoidal TMDSC analysis was undertaken on 8.3 mg of PCL-blend sample sealed within an aluminum pan and heated at 0.5 °C/min under a constant purge of inert N_2_ gas.

#### 2.4.2. Porosimetric Evaluation of the 3D Printed PCL Scaffold

Porosimetric analysis of the 3D printed PCL blend and its polymeric constituents was performed using an ASAP 2020 porositometer (Micromeritics Instrument Company (Pty) Ltd., Norcross, GA, USA) equipped with research grade ASAP 2020 V3.01 software (*n* = 3). This was employed to determine the quantifiable aspects of the polymers porous nature, such as the surface area, pore size diameter, and total pore volume. Briefly, the pieces of the PCL samples (20 mg) were evacuated with nitrogen gas (N_2_) (hard-sphere diameter = 3.860 Å; molecular cross-section = 0.162 nm^2^) for the removal of surface moisture and gas particles prior to analysis. All samples were thereafter covered in an isothermal jacket and degassed for approximately 18 h. The degassed samples were then transferred to the analysis port and immersed in liquid nitrogen prior to the porosity analysis. The Barrett–Joyner–Halenda (BJH) and Brunauer–Emmett–Teller (BET) adsorption and desorption relationships were subsequently generated.

### 2.5. Preparation of the PVA–PAA Hydrogel Containing Hydrophilic Sodium Indomethacin Scaffolds

The PVA–PAA hydrogel containing sodium indomethacin was prepared by mixing 200 mg indomethacin in 20 mL 6% PVA-NaOH solution on a magnetic stirrer. Acrylic acid (1 mL) was then added to the solution and mixed for 1 min. *N*,*N*-Methylenebisacrylamide (100 mg) was thereafter added to the mixture and mixed for a further 2 min to form a homogenous solution. The solution was allowed to stand to attain a medium viscosity prior to 3D printing. The 3D printing of the PVA–PAA hydrogel was attained by adding the 20 mL PVA–PAA hydrogel to a low temperature printing cartridge with a plastic tip maintained at 25 °C. The PVA–PAA hydrogel was then printed into the free surface area octagonal structure of the PCL scaffold at 2.0 mm/s at 3.5 bar to form the PCL–PVA–PAA (PPP) scaffold. The PPP scaffolds were then left at room temperature for 24 h prior to evaluation.

**Figure 2:**
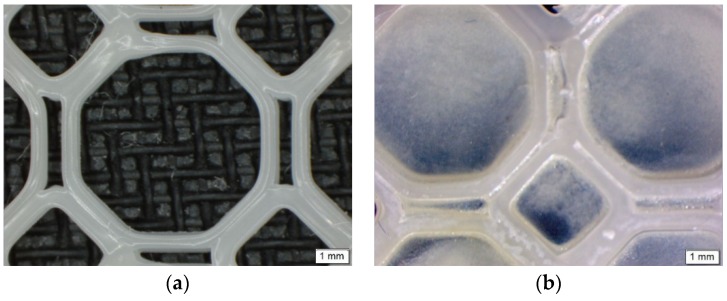
Microscopic images of (**a**) the 3D printed PCL scaffold and (**b**) the PPP scaffold highlighting uniform adhesion of the PVA–PAA hydrogel within the octagonal structure.

Imaging of the PCL scaffold and the PPP scaffold (Figure 2) were undertaken using an Olympus SZX 7 automated microscope equipped with Olympus Stream Basic V1.9.4 imaging software (Olympus Soft Imaging Solutions GmbH, Münster, Germany). All images were taken using a MPLAPON 1× objective lens at a 0.5× magnification and 81,000 µm objective working distance. The pixel size for the images were set to 2560 × 1920 with the brightness and saturation set to 0, the contrast set to 1.00 and the sharpness set to 5.00.

#### 2.5.1. Viscoelastic Assessment of the PVA–PAA Hydrogel

The viscoelasticity of the PVA–PAA hydrogel was determined using an Elasto-SensTM Bio (Rheolution, Montreal, Canada) viscoelasticity analyzer (*n* = 3). Briefly, after calibration of the instrument, 3 mL of liquid PVA–PAA solution was placed within the sample holders and allowed to form a hydrogel. Phosphate buffered saline (PBS) (pH 7.4) was then added to the sample holder prior to one drop of dispersant oil. The samples were then placed within the thermal chamber of the instrument and run for 8 h at 37 °C with a measurement rate of 15 min, and a 5 min soak time. The storage modulus (G′), loss modulus (G″), tan δ and sample height were determined over the analysis period. The PVA–PAA hydrogel samples were run in triplicate.

#### 2.5.2. Structural Validation of the PCL Blend and PVA–PAA Hydrogel

Fourier transform infrared spectroscopy (FTIR), utilizing a Perkin Elmer Spectrum 2000 FTIR spectrometer equipped with a MIRTGS detector (PerkinElmer Spectrum 100, Llantrisant, Wales, UK), was undertaken to detect the vibration characteristics of the chemical functional groups in the PCL blend, before and after printing, as well as its native polymers. The respective PCL and PVA–PAA hydrogel samples were prepared as pellets against a blank background and analyzed at a wave number ranging from 4000–650 cm^−1^ at a resolution of 4 cm^−1^ for 10 scans. Deconvolution of the generated FTIR spectra was further carried out to determine variations in the PCL blend structure in relation to its native polymers.

#### 2.5.3. Magnetic Resonance Imaging of the PPP Scaffold in Simulated Physiological Media

A magnetic resonance imaging system, with digital MARAN-i System configured with a DRX2 HF Spectrometer console (Oxford Instruments Magnetic Resonance, Oxon, UK), equipped with a compact 0.5 Tesla permanent magnet stabilized at 37 °C and a dissolution flow through cell was employed for viewing of the swelling behavior of the PPP scaffold. After duly configuring, optimizing the shims and probe tuning, the cone-like lower part of the cell was filled with glass beads to provide a laminar flow at 16 mL/min. of the solvents employed. A 3D printed scaled-up PPP cell was then placed in position within the cell, which in turn was positioned in a magnetic bore of the system, with the magnetic resonance images acquired every 10 min with MARAN-i version 1.0 software.

#### 2.5.4. Mechanical Strain Analysis of the PCL and PPP Scaffolds

**Figure 3:**
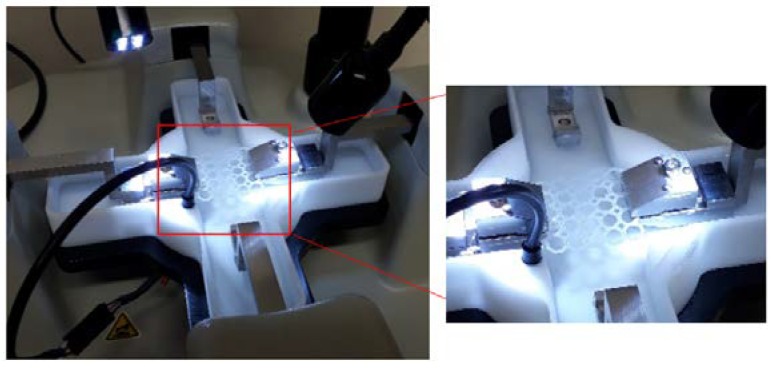
Equipment configuration for the uniaxial strain testing of the PCL scaffold in PBS (7.4, 37 °C).

Uniaxial tensile strain testing was conducted on the PCL scaffolds and PPP scaffolds using a BioTester 5000 Biomaterials Tester (CellScale, Waterloo, ON, Canada) equipped with 5 N load cells (*n* = 3) in accordance with an ASTM standard protocol (D882-02). The scaffold samples were attached onto the stainless-steel clamping mechanisms (Figure 3) and suspended in PBS (pH 7.4; 37 °C) to assess scaffold displacement, rigidity, and strength under simulated physiological conditions of pH and temperature. Prior to mounting of the scaffold, the Biotester was balanced, calibrated, and moved to 3200 mm apart. Once mounted, the samples were subjected to a preload of 20 mN and thereafter 5 N of strain was applied as a ramp force until displacement had plateaued to simulate prolonged strain. The scaffolds were thereafter set to recover over a period of 10 s with the procedure repeated for two additional runs. The scaffolds were thereafter evaluated for breakage, peak size, peak displacement, and peak force. Stress-strain graphs were thereafter constructed from the generated data with the stress-strain modulus of the scaffold thereafter determined. The analysis was repeated on anhydrous PCL scaffolds to ascertain the effect of hydration on scaffold strength. Uniaxial strain testing was also undertaken on the additional geometrical orientations with the results detailing the octagonal structure having the greatest resistance to strain (Supplementary Materials S1). 

#### 2.5.5. In Vitro Sodium Indomethacin Release Analysis of the PPP Scaffold

In vitro drug release analysis (*n* = 3) was undertaken to determine the rate of release of sodium indomethacin from the PPP scaffolds. The scaffold samples containing 50 mg sodium indomethacin were placed in 20 mL of PBS (pH 7.4) maintained at 37 °C in a shaker bath rotating at 30 rpm. The analysis was undertaken over 8 h with samples (1 mL) removed at 30, 60, 90, 120, 180, 240, 300, 360, 420, and 480 min and analyzed by UV spectroscopy (Lambda 25, UV–vis spectrometer, PerkinElmer^®^, Waltham, MA, USA) at 320 nm. Fresh buffer (1 mL) was replaced at each sampling point to maintain sink conditions. The sodium indomethacin concentration in the drug release media was thereafter calculated using a preconstructed calibration curve (ε = 1.707) at 320 nm. To determine the effect of swelling on the release of sodium indomethacin, the PVA–PAA hydrogel, dried at 25 °C for 24 h, was evaluated for change in gel height in response to exposure to drug release media using the viscoelastic assessment stated earlier.

## 3. Results

### 3.1. In Vitro Characterization of the PCL Blend

#### 3.1.1. Thermal Profiling of the PCL Blend

**Figure 4:**
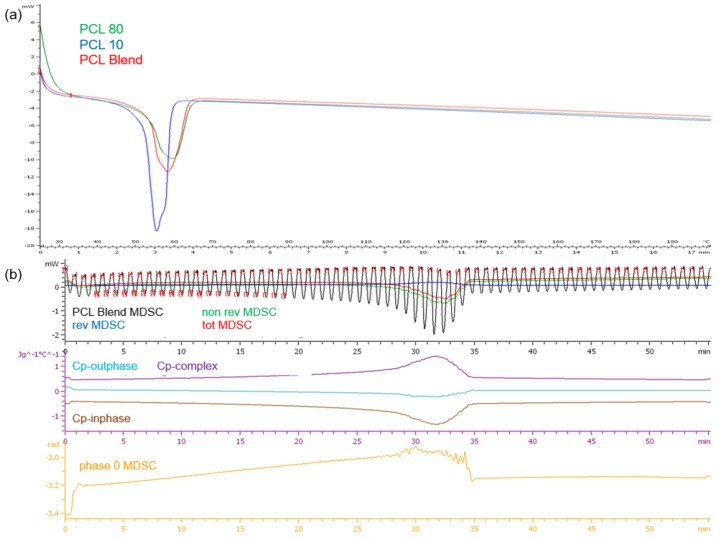
Thermograms of (**a**) the PCL_10_, PCL_80_ and PCL blend DSC profiles and (**b**) the TMDSC profile of the PCL blend.

DSC analysis of the PCL polymers revealed single melting endotherms in all thermograms (Figure 4a). PCL_10_, having a lower molecular weight than PCL_80_, had a lower melting point temperature (T_m_) at 54.49 °C compared to PCL_80_ at 58.88 °C, the PCL blend had a T_m_ of 57.81 °C. It has been demonstrated that copolymers generally show lower crystallization temperatures and melting than the homopolymers. The narrow range in temperature over which melting occurs displays the differing lengths of the chains in the polymer samples. Evaluation of the PCL blend TMDSC profile (Figure 4b) revealed that the endothermic transition occurred at approximately 32 min or 51 °C, marginally lower than the DSC profile. It was also noted that there was a decrease in cp-inphase with cp-outphase remaining at the baseline. Cp-complex was however seen to increase. The phase 0 curve was also seen to increase with an increase in temperature, but returned to baseline after melting with no fluctuations over the test period. This result therefore validated the thermal stability of the PCL blend once melting had occurred.

#### 3.1.2. Porosimetric Assessment of the 3D Printed PCL Scaffold

**Figure 5:**
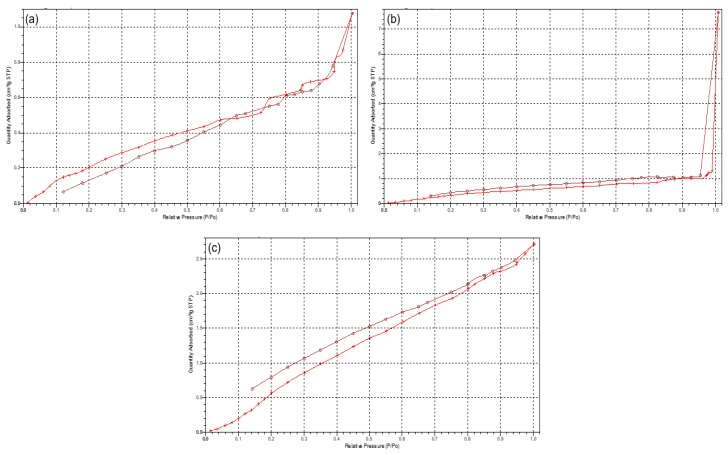
Typical isotherms of (**a**) PCL_10_, (**b**) PCL_80_, and (**c**) 3D printed PCL blend.

Due to the scaffolds being composed of PCL, a low-degrading polymer, the porous nature of this scaffold can allow for a greater structural integrity and robustness. However, as the drug-loaded hydrogel system has been incorporated onto the surface of the PCL scaffold, the primary functionality of the PCL pores would therefore be for the enhanced proliferation and adhesion of the drug loaded hydrogel. Results of the porosity studies undertaken (*n* = 3) indicated a reversible Type III isotherm for PCL_10_ and the PCL blend [17], explaining the formation of a multilayer in the polymer melt (Figure 5). The PCL_10_ polymer was limited to an uptake of 1.25 (±0.07) cm^3^/g due to the hindrance of the multilayer nature of the polymer. The pore size of PCL_10_ (46.768 Å ± 1.266) was also seen as lower than that of PCL_80_ (51.299 Å ± 1.810). Porosity analysis of PCL_80_ displayed a Type I isotherm, indicative of microporosity [17], with the greatest adsorption uptake of 7.85 (±0.501) cm^3^/g obtained. The 3D printed PCL blend had the lowest pore size (43.001 Å ± 1.610), with an uptake of 2.7 (±0.037) cm^3^/g. Additionally, imaging of the PCL scaffold (Figure 2) revealed no distinct macroporosity for enhanced hydrogel adhesion, ensuring that the PCL microporosity had the greatest effect in allowing for the strong adhesion of the PVA–PAA hydrogel onto the PCL scaffold surface.

### 3.2. In Vitro Characterization of the PVA–PAA Hydrogel

#### Viscoelastic Evaluation

**Figure 6:**
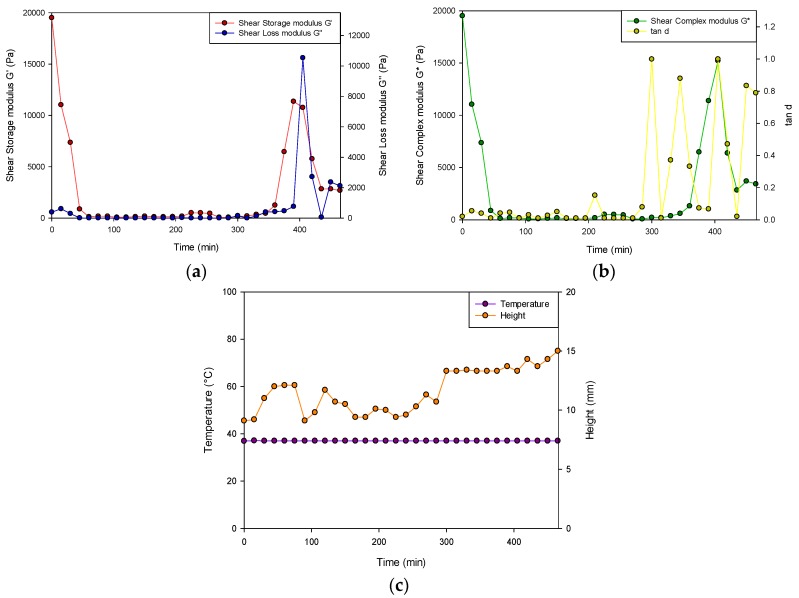
Viscoelastic profiles of the PVA–PAA hydrogel where (**a**) represents the shear storage modulus (G′; SD ≤ 352 Pa in all cases) and shear loss modulus (G″; SD ≤ 520 Pa in all cases), (**b**) represents the shear complex modulus (G*; SD ≤ 265 Pa in all cases) and tan δ of the hydrogel (SD ≤ 0.088 in all cases) and (**c**) depicts the height of the hydrogel (SD ≤ 1.2 mm in all cases) and temperature of the test over the 8-h test period (SD ≤ 0.0 °C in all cases).

Figure 6 represents the change in sample height (mm), the shear storage modulus (G′), the shear loss modulus (G″), the complex modulus (G*) and the loss tangent (tan δ) after 8 h of gelation at 37 °C (*n* = 3). At that beginning of the analysis, G′ was larger (19510 ± 352 Pa) than G” (390.2 ± 12.1 Pa) therefore determining that the hydrogel behaved as a semi-solid. Consequently, at 400 min, G” was greater than G′, indicating that a less solid-like behavior was dominant in the hydrogel.

As the analysis proceeded, tan δ as well as the shear complex modulus (G*) approached 1.00, stating that the hydrogel was less viscous and therefore fully hydrated with an appreciable resistance to deformation. The tan δ, which was determined to increase after 300 min, did not exceed 1 for the remainder of the study, therefore validating that it remained as a hydrogel and not as a liquid state upon hydration. Buffer solutions generally have a low ionic strength with the absorption of few of the present mobile ions required to balance the osmotic pressure thus facilitating swelling. Steric interactions among the charged polymer groups occur inside the hydrogel due to its low ion concentration, therefore promoting increased hydrogel swelling due to the osmotic gradient formed. The test sample additionally underwent fluctuations in height (9.2–15 mm) over the test period. The temperature of the study also remained consistent at physiological conditions.

### 3.3. In Vitro Characterization of the PPP Scaffolds

#### 3.3.1. Synthesis Validation of the PCL Scaffold and PVA–PAA Hydrogel

**Figure 7:**
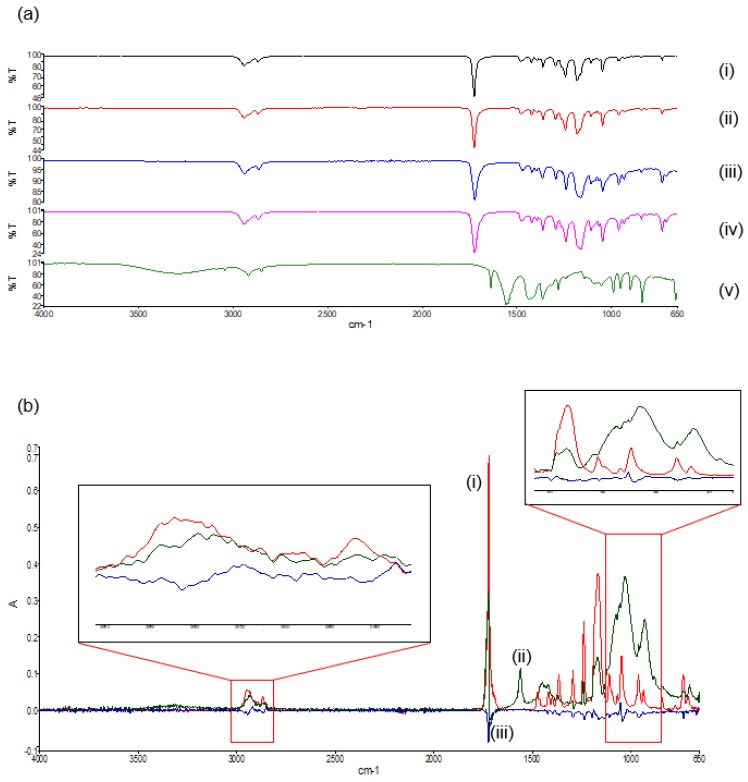
FTIR spectra of (**a**) the transmittance profile of (i) PCL_10_,(ii) PCL_80_, (iii) the PCL blend, (iv) the 3D printed PCL blend, and (v) the PVA–PAA hydrogel; and (**b**) the deconvolution absorbance profile of (i) PCL_10_, (ii) PCL_80_, and (iii) the PCL blend.

In the FTIR spectra of the native PCL polymers and their derivatives (Figure 7a), bands can be seen at 2942 cm^−1^, indicative of asymmetric CH_2_ stretching. The prominent, strong bands seen around 1725 cm^−1^ are representative of carbonyl stretching [18]. The carbonyl stretching in the polymer blend has a reduced intensity in comparison to the native polymers, as a result of modification due to the melt dispersion. At 1110 cm^−1^, C–O and C–C stretching in the amorphous phase of the blend can be seen, allowing for a less rigid polymer [19]. The spectra of both the polymer blend and the 3D printed polymer blend indicated no substantial shift in the band frequencies as compared to the native polymers, concluding that the melting and the 3D printing of the native polymers did not significantly chemically modify the polymers or disrupt the chemical orientation. The FTIR spectra of the PVA–PAA hydrogel revealed a peak at 2919 cm^−1^, indicative of C-H stretching pertaining to PVA [20]. The 1537.06 cm^−1^ peak is attributed to NH_2_ deformation. Additionally, the peaks around 1636.4 cm^−1^ can be assigned to the amide I bands due to cross-linking by *N*,*N*′-methylenebisacrylamide [21].

Deconvolution of the FTIR spectra detailed variations in absorbance between the PCL blend and its native polymers. Evaluation of the deconvolution profile (Figure 7b) determined noticeable decreases in absorbances of the PCL blend between 1238 cm^−1^ and 853 cm^−1^. This was again seen between 2994 cm^−1^ and 2830 cm^−1^. This result confirmed that even though no additional peaks were seen in the FTIR transmittance profiles, decreases in the absorbance values between the PCL blend and the PCL_10_ and PCL_80_ was a result of a chemical blend being formed.

#### 3.3.2. Mechanical Strain Assessment of the PCL and PAA Scaffolds

**Figure 8:**
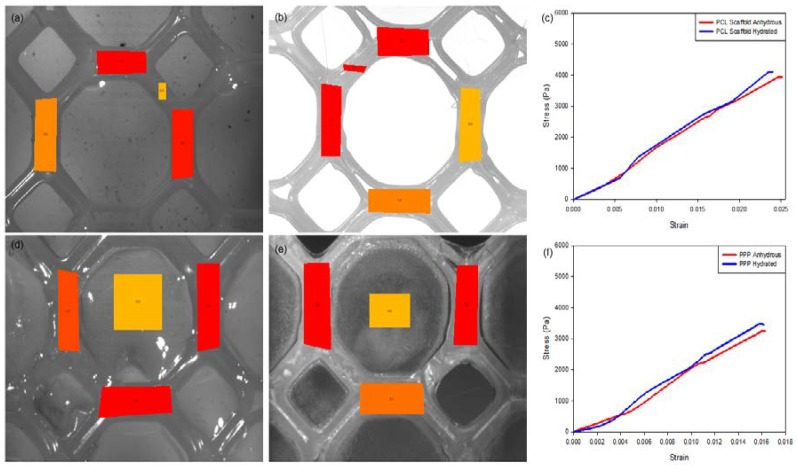
Strain-tracking images of (**a**) the hydrated PCL and (**b**) the anhydrous PCL. (**c**) Strain graph of the PCL scaffold. Strain-tracking images of (**d**) the hydrated PPP and (**e**) the anhydrous PPP scaffold. (**f**) Strain graph of the PPP scaffold. Degree of strain exerted at specific points on the scaffold is represented in red (greatest), orange (intermediate) and yellow (least) strain.

Mechanical strain evaluation of the PCL scaffolds without the added PVA–PAA hydrogel revealed minimal differences between the hydrated and anhydrous scaffold samples (Table 1; *n* = 3). Results of this analysis (Figure 8) determined that the hydrated PCL scaffold had a greater resistance to strain with a maximum displacement of 893 (±42) µm and a modulus of 174.51 (±6.72) KPa compared with the anhydrous scaffold which had a maximum displacement of 1047 (±45) µm and a modulus of 166.21 (±6.01) KPa. This result can be attributed to the elastic nature of anhydrous PCL with its mechanical properties changed due to hydration of the PCL structure [22,23]. This result was further validated through strain tracking of the PCL scaffold (Figure 8a) where a distribution of strain of maximum 4.5 (±0.2) was noted on the hydrated scaffold sample compared to the anhydrous sample with a maximum strain of 5.1 (±0.2). Repetition of the strain analysis revealed limited variation in the maximum displacement and modulus of the PCL scaffold detailing its ability to resist strain under repetitive applications of force.

A similar result was determined for the PPP scaffolds containing the PVA–PAA hydrogel (Table 1, *n* = 3). Strain analysis of the hydrated PPP scaffold (Figure 8b) noted a maximum displacement of 464 (±32) µm and a modulus of 219.84 (±9.22) KPa for the scaffold with a maximum strain of 5.6 (±0.3) determined during strain tracking. Comparatively, the anhydrous PAA scaffold was determined to have a maximum displacement of 633 (±38) µm and a modulus of 202.04 (±9.05) KPa with a maximum strain of 8.6 (±0.5) noted during strain tracking. The large difference in strain tracking results between the PCL and PPP scaffolds can be attributed to the tracking system analyzing the weaker PVA–PAA hydrogel on the surface of the PCL scaffold framework. This result is also in correlation with the PCL scaffold, where the hydrated scaffold was determined to have a higher modulus compared to the anhydrous PCL scaffold. It is also noted that the inclusion of the PVA–PAA hydrogel significantly increased the strength of the PCL scaffold, contributing to its potential as a strain-resistant reinforcement platform. 

#### 3.3.3. Magnetic Resonance Imaging of the PPP Scaffold

**Figure 9:**
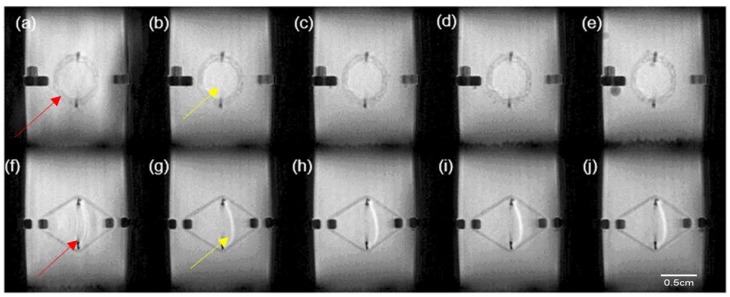
MRI images of the PPP scaffold from the front view at (**a**) 0 h; (**b**) 2 h; (**c**) 4 h; (**d**) 8 h; (**e**) 24 h and from side view at (**f**) 0 h; (**g**) 2 h; (**h**) 4 h; (**i**) 8 h; and (**j**) 24 h, where red arrows depict the PCL scaffold and yellow arrows depict the PVA–PAA hydrogel.

MRI results of the PPP scaffold over the 24-h analysis period revealed minimal swelling of the PCL matrix correlating to the viscoelastic analysis results achieved (Figure 9). This result validated the use of the PCL blend for the development of the PPP scaffold. Evaluation of the PVA–PAA hydrogel in the PPP scaffold revealed minimal change in the hydrogel matrix size with maximum swelling achieved within the first 2 h of the study. The minimal swelling of the PVA–PAA hydrogel can be attributed to the formation of carboxylic anions in the hydrogel network, resulting in the decreasing of osmotic pressure between the hydrogel matrix and the analysis media [24]. It was also determined that the hydrogel shape and size remained consistent over the 24-h test period. Additionally, it was also noted that the PVA–PAA hydrogel remained on the scaffold surface over the test period. This result therefore revealed the successful incorporation of the PVA–PAA blend onto the scaffold matrix allowing for it be utilized as a drug delivery system at the site of implantation.

#### 3.3.4. Sodium Indomethacin Release Profiling of the PPP Scaffold

**Figure 10:**
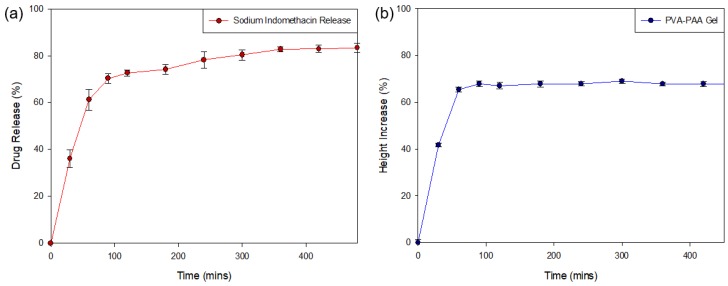
Drug release profile of (**a**) sodium indomethacin from the PPP scaffold (SD ≤ 4.36% in all cases) with (**b**) the change in height of the PVA–PAA hydrogel in response to exposure to simulated drug release media (0% representing the gel height at time 0 mins; SD ≤ 1.41% in all cases).

Analysis of the release of sodium indomethacin from the PPP scaffold (Figure 10a) was determined over the 8-h (480 min) test period (*n* = 3). Evaluation of the generated drug release profile revealed an initial burst release of weakly-bound highly hydrophilic sodium indomethacin (41.12 ± 3.81%) from the PPP scaffold within the first 30 min, with 65.23% (±4.36) released in the first hour. This result was in correlation with the MRI swelling data that determined that maximum matrix swelling occurred within the first 2 h. Evaluation of the PPP over the 8-h (480 min) test period revealed the sustained release of sodium indomethacin from Hour 2 (60 min) to Hour 8 (480 min) with an 83.36% (±1.88) drug release after 8 h. This release profile was in correlation to the change in gel height profile (Figure 10b), where a rapid increase in height of the dry PVA–PAA gel was seen in the first 90 min, correlating with the burst release of sodium indomethacin noted. The height of the gel remained consistent from Hour 2 until the end of the study thereby explaining the transition to a sustained release profile where sodium indomethacin release was primarily by diffusion. This positive drug release result therefore confirmed that exposure of the PPP scaffold to simulated physiological media can be highly beneficial in conditions where inflammation occurs. The initial rapid and subsequent sustained release of sodium indomethacin can also be beneficial in biological tissue where prolonged healing is needed. Whilst, the prepared PPP scaffold provides for a large proportion of drug release in the first 8 h, this platform can be modified to allow for prolonged release of drugs in conditions which require it.

## 4. Discussion

PCL has been noted in previous studies to be a highly suitable polymer for 3D printing. Its hydrophobic nature, resistance to strain, and ability to erode slowly over time further allows this polymer to be used effectively as a strain-resistant reinforcement platform for drug release systems. The synthetic polymer blend prepared in this study and used in the fabrication of the PCL scaffolds has been determined to 3D print reproducibly and significantly quicker at a lower pressure when compared to the previously utilized PCL (Mw 48,000–90,000) [13,25,26]. This property therefore allows for a more effective timeous 3D printing process.

Evaluation of the thermal, porosimetric, and structural properties of the PCL blend also provided important information on the blend which was compared to its native constituents. While no manipulation of the individual PCL matrices was determined from the combination of the different PCL molecular weights, these analyses provided data on the effects of the printing process on the PCL blend. FTIR transmittance analysis determined that the PCL blend had no additional peaks when compared to its native constituents, validating that the PCL_10_ and PCL_80_ structures had been maintained throughout the printing process. FTIR deconvolution, however, determined decreases in absorbance of the PCL blend when compared to the native polymers. This result therefore determined that a chemical interaction did occur in the formation of the blend, however, with minimal variation from its native constituents. Additionally, results determined in the thermal analysis of the PCL blend revealed a melting point temperature of 57.81 °C with no significant variations between the PCL blend at its native constituents. MDSC analysis revealed the thermal characteristics of the PCL blend, with results also determining its thermal stability over the test period.

Evaluation of the generated porosity data noted properties of the scaffold that would be beneficial for inclusion of drug-loaded hydrogel systems. The printed PCL blend was seen to have the lowest pore volume (43.001 Å), with an uptake of 2.7 cm^3^/g. This can be due to the pressure applied to the PCL blend in its molten state during the printing process, decreasing the size and volume of the surface pores. The results of this study however can be seen to be within an acceptable range for application as a drug delivery-structural support system.

Strain testing of the 3D printed PCL and PPP scaffolds determined the robustness and resistance of the scaffolds to prolonged and continuous strain, which is essential for load-bearing tissue-support scaffolds. The addition of the PVA–PAA hydrogel to the PCL scaffold was furthermore determined to increase the structural strength of the scaffold. This result was significant as it determined that the prepared 3D printed scaffolds act both as a drug delivery platform as well as a reinforcement matrix that can provide structural support to damaged or repaired biological tissues. The degree of rigidity and strength required in the reinforcement of these tissues can be modified through varying of thickness of the PCL scaffolds in addition to modification of the geometrical orientations to the extension requirements of the load-bearing tissue (Supplementary Data).

Evaluation of the PPP scaffold further determined that the prepared drug delivery hydrogel system was successfully 3D printed onto the PCL-blend platform with the slow gelation properties of the PVA–PAA solution ideal for adhesion of the 3D printed solution to the sides of the octagonal PCL structure. This adhesion was promoted by the adhesive nature of the PVA–PAA hydrogel in combination with the porous nature of the PCL scaffold. FTIR analysis of the PVA–PAA hydrogel revealed structural properties expected of acrylic acid hydrogels, detailing that the 3D printing process did not have any structural effects on the PVA–PAA matrix. MRI and viscoelasticity studies undertaken further determined the matrix absorption and mechanical properties of the prepared PVA–PAA hydrogel which was determined to have rapid swelling characteristics as well as matrix robustness. Additionally, sodium indomethacin release from the PVA–PAA hydrogel was determined to be initially rapid with a sustained release profile thereafter. This can be expected due to the weakly bound, hydrophilic properties of the sodium indomethacin used. Variations in the PVA–PAA composition as well as in the drug utilized, provides for the prepared PPP scaffold to be advanced further for other drug delivery applications. 

The in vitro results of this study therefore confirm the ability of the PPP scaffold for application as a potential structural support structure in load-bearing tissue damage repair. The generated release profile of sodium indomethacin from the PPP scaffold further validates its ability for rapid drug release in simulated physiological conditions of temperature and pH. As the current treatment of injured load-bearing tissue involves suturing, the usage of structural support scaffolds with therapeutic actions will assist in a more effective treatment of the damaged tissue, thereby minimizing rehabilitation of the damaged area and improving patient care. With the positive results achieved in this study, the prepared PPP scaffold is proposed as a suitable candidate to be advanced into an optimized tendon and ligament structural support scaffold in accordance with the designated standards, to undergo ex vivo studies utilizing excised tendon tissue as well as further biocompatibility, toxicology, and in vivo studies to determine its effectiveness in enhancing load-bearing tissue damage repair.

## 5. Conclusions

This study provides an interesting application of a 3D printed polymeric platform for the structural support of damaged load-bearing tissue in addition to being utilized as a drug delivery system. The positive results displayed in the preparation and characterization of the PCL-blend highlighted its quick, reproducible 3D printing properties as well its thermal stability and pore size and volume for inclusion of drug-loaded hydrogel systems. Strain testing of the 3D printed PPP scaffolds further highlighted its ability to be used as a reinforcement platform for load-bearing tissue support whilst also providing sodium indomethacin for treatment of inflammation at the implantation site. These results therefore support the use of the prepared PPP scaffold system for the treatment and reinforcement of load-bearing tissue injury. 

## Figures and Tables

**Table 1:** Results of the mechanical strain assessment of the PCL and PPP scaffolds.

Scaffold	Anhydrous		Hydrated	
	Maximum Displacement (µm)	Modulus (KPa)	Maximum Displacement (µm)	Modulus (KPa)
PCL	1047 (±45)	166.21 (±6.01)	893 (±42)	174.51 (±6.72)
PPP	633 (±38)	202.04 (±9.05)	464 (±32)	219.84 (±9.22)

## Supplementary Materials

The following are available online at http://www.mdpi.com/1996-1944/11/6/1006/s1, Figure S1: 3D designs of the PCL-blend geometrical scaffolds comprising of (a) circles, (b) octagons and (c) squares with their corresponding images with an inner structure and without an inner structure respectively, Table S1: Weight, free surface volume, moisture uptake and modulus of the respective 3D printed PCL geometrical scaffolds.
