# Supplementary material for: 3D Printed, PVA–PAA Hydrogel Loaded-Polycaprolactone Scaffold for the Delivery of Hydrophilic In-Situ Formed Sodium Indomethacin
Source: Materials (Basel). 2018 Jun 13;11(6):1006. doi: 10.3390/ma11061006 (PMC6024948; doi:10.3390/ma11061006)
Supplement: Supplementary file 1 [file materials-11-01006-s001.pdf]

Supplementary Materials

3D Printed, PVA–PAA Hydrogel Loaded-Polycaprolactone Scaffold for the Delivery of Hydrophilic In-Situ Formed Sodium Indomethacin

Mershen Govender, Sunaina Indermun, Pradeep Kumar, Yahya E. Choonara and Viness Pillay *

Wits Advanced Drug Delivery Platform Research Unit, Department of Pharmacy and Pharmacology, School of Therapeutic Sciences, Faculty of Health Sciences, University of the Witwatersrand, 7 York Road, Parktown, Johannesburg 2193, South Africa; mershen.govender@wits.ac.za (M.G.); sunaina.indermun@wits.ac.za (S.I.); pradeep.kumar@wits.ac.za (P.K.); yahya.choonara@wits.ac.za (Y.E.C.)

* Correspondence: viness.pillay@wits.ac.za; Tel.: +27-11-717-2274

S1. Methods

S1.1. Design and printing of the PCL-blended geometrical scaffolds

The PCL-blended geometrical scaffolds were designed using Magics ® V18 design software equipped with Envisiontec® printing protocols (Figure S1a-c). The scaffolds were thereafter printed using an Envisontec® 3D Bioplotter (Envisontec® GmbH, Gladbeck, Germany) as described previously. The printing procedure was undertaken both with and without an inner structure to ascertain its effect on scaffold rigidity and free surface area. The inner structure was set to a 90° orientation spread 0.6 mm apart.

S2. Results and Discussion

S2.1. Mechanical and Swelling Properties of the 3D printed PCL geometrical scaffolds

Evaluation of the 3D printed PCL octagonal scaffolds detailed significant differences in the weights and mechanical properties of the scaffolds with and without the inner structure (Table S1). The weights of the individual scaffolds varied greatly with the square orientation with an internal structure having the greater mass and thus greater PCL content (282.4 mg). The octagon geometry without an internal structure was determined to have the lowest mass with 201.6 mg. The octagon orientation with an internal structure was determined to have a mass of 242.5 mg. Interestingly, there was a limited variation in PCL content between the inner structure and no inner structure groups for the circular geometry when compared to the other 2 geometries detailing that the inner structure of the circular arrangement is minimal when compared to the octagon and square arrangements. Moisture uptake or swelling of the scaffolds were uniform across all the prepared scaffolds due to the common constituents of the scaffolds. A limited variation was noted with a standard deviation of 0.057 calculated between all the prepared scaffold arrangements.

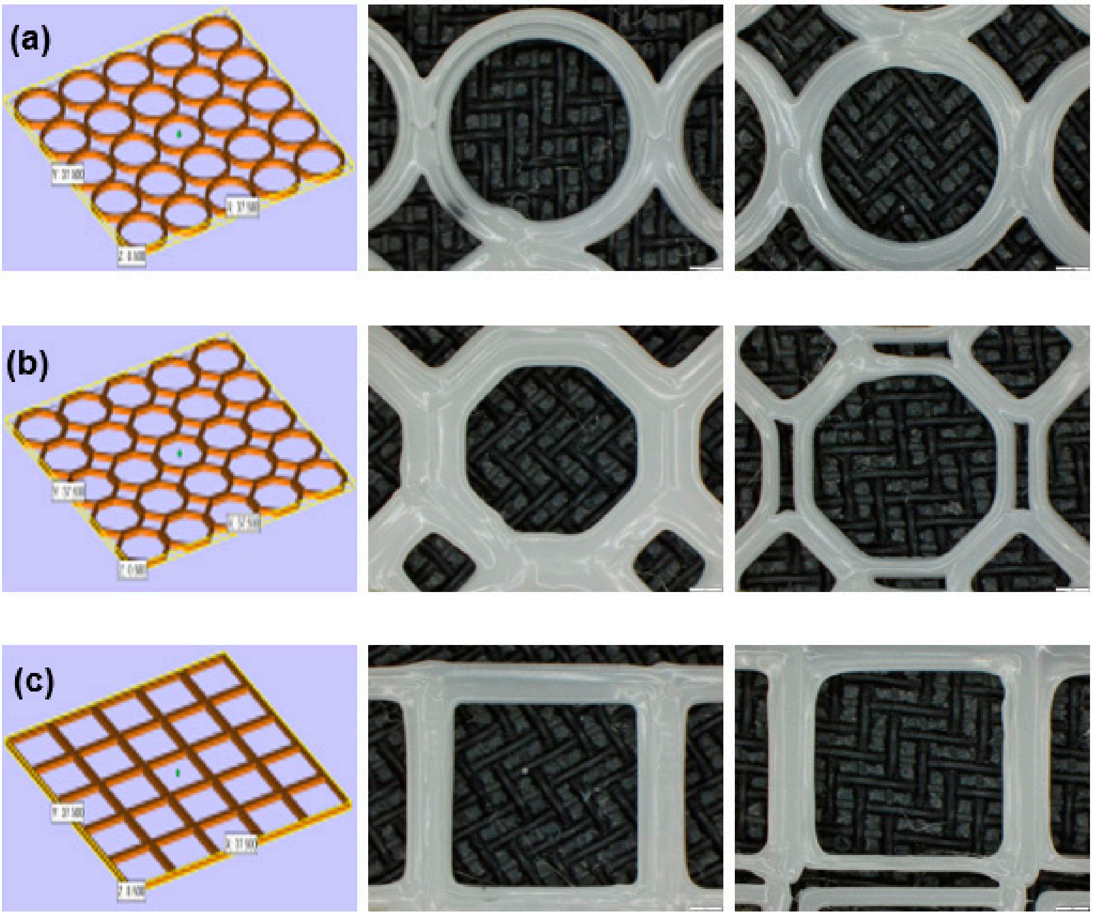

Figure S1. 3D designs of the PCL-blend geometrical scaffolds comprising of (a) circles, (b) octagons and (c) squares with their corresponding images with an inner structure and without an inner structure respectively.

Uniaxial strain testing of the PCL scaffolds detailed contrasting results between each geometric orientation (Table S1). The hydrated square arrangement with an internal structure was noted to have the greatest resistance to strain with a Modulus of 499.01 KPa. This can be attributed to the more pronounced triangular internal structure printed within this arrangement when compared to the other orientations resulting a greater resistance to strain. The square orientation was followed by the octagon arrangement with an internal structure with a Modulus of 388.01 KPa. The circular arrangement had the lowest resistance to strain with a Modulus of 167.77 KPa. In all arrangements, the inner structure group had a lower Modulus of 150.05 KPa, 174.51 KPa and 189.09 KPa for the circular, octagon, and square arrangements respectively. The square arrangement with no internal structure was also noted to have no defined shape with the arrangement breaking apart upon application of the preload. The subsequent resistance to strain determined was as a result of the outer structure of the scaffold remaining with the inner square cells breaking apart.

Uniaxial strain testing of the anhydrous 3D printed PCL scaffolds also revealed contrasting results. The octagon arrangement was noted to have the greatest resistance to strain in the anhydrous group with a Modulus of 208.50 KPa and 166.21 KPa for the inner structure and no inner structure groups respectively. The was a minimal difference between the circular arrangement with no inner structure and the square arrangement with an internal structure. It was also determined that there was a greater resistance to strain once each of the scaffolds were hydrated. This can be attributed to the brittle PCL_{10} content of the PCL blend that resulted in fracture points within the structure of the scaffolds that was overcome once the matrix had been hydrated. Analytical evaluation of the free scaffold volume (Table S1) detailed that the octagon-shaped network had the greatest free scaffold volume of 534.9 mm^3 and 584.5 mm^3 for the internal structure and no internal structure groups

respectively. The circular and squared-shaped networks were noted to have a weigh and free scaffold volume of 223.9 mg and 665.3 mm^3 and 221.7 mg and 471.6 mm^3 respectively. on with the no inner structure group. Due to the positive results achieved with the octagon scaffold, this geometrical orientation was chosen to be advanced into a drug delivery platform due to its rigidity, decreased polymeric weight, resistance to strain and free surface area for inclusion of the hydrogel.

Table S1. Weight, Free surface volume, Moisture Uptake and Modulus of the respective 3D printed PCL geometrical scaffolds.

**Geometrical scaffold**	**Weight (mg)**	**Free surface volume (mm^3)**	**Moisture Uptake (%)**	**Modulus Hydrated (KPa)**	**Modulus Anhydrous (KPa)**
Circle (I)	224.1	664.9	1.10	167.77	127.86
Circle (NI)	223.9	665.3	1.12	150.05	147.19
Octagon (I)	242.5	534.9	1.02	388.01	208.50
Octagon (NI)	201.6	584.5	1.09	174.51	166.21
Square (I)	282.4	471.6	1.15	499.01	141.84
Square (NI)	221.7	447.6	1.01	129.09	109.36

I = Inner structure; NI = No inner structure
